# O05 Resistance on enteric surveillance of children is an effective predictor of resistance in Gram-negative bloodstream infections in a two-centre study

**DOI:** 10.1093/jacamr/dlaf230.005

**Published:** 2025-12-04

**Authors:** Andrew McArdle, Victoria Austin, Seán Whelan, Alexander Brown, James Hatcher

**Affiliations:** Alder Hey Children’s Hospital, Liverpool, UK; Alder Hey Children’s Hospital, Liverpool, UK; Children's Health Ireland, Ireland; Institute of Child Health, University College London, London, UK; Great Ormond Street Hospital, London UK

## Abstract

**Background:**

Routine surveillance for resistant organisms in hospitalized patients’ gastrointestinal tracts is used to prevent spread. Results can also be used to inform empirical antibiotic recommendations. However, the association between enteric resistance on surveillance and resistance in subsequent invasive infections in children is not evidenced

**Objectives:**

We studied the capacity of resistance identified from enteric surveillance to predict resistance in Gram-negative bloodstream infection isolates (GN-BSII).

**Patients and methods:**

We obtained respectively 10 years and 7 years 8 months of data from Great Ormond Street and Alder Hey Children’s Hospitals (GOSH and AHCH, respectively), comprising GN organisms identified on enteric surveillance and blood cultures. For each unique GN-BSII with surveillance result available in the preceding 5 days to 6 months, the presence of resistance to specified antibiotics was determined. 2×2 tables were constructed for each antibiotic. Within BSII with surveillance available in the preceding 5 days to 1 year, changes in test performance with increasing lookback were explored. Sensitivity analyses explored approaches with organism matching and application of EUCAST rules. Organism-group-weighted resistance probability predictions were produced from the organism matching approach.

**Results:**

A total of 1119 individuals had GN-BSII, of which 91% (*n*=1022) had surveillance available. 1830 isolates were included. *Escherichia coli*, *Klebsiella pneumoniae*, *Enterobacter* spp. and *Pseudomonas aeruginosa* represented 56% of BSII. 53% and 77% of patients (AHCH and GOSH) had surveillance in the prior 6 months. Rates of resistance to gentamicin, piperacillin/tazobactam and ciprofloxacin were higher in BSII from patients with preceding resistance on surveillance samples than in patients without in GOSH (36% versus 7%, 36% versus 15%, 46% versus 13%; PPV versus NPV) and AHCH (38% versus 9%, 36% versus 15%, 31% versus 10%). While meropenem resistance was uncommon in AHCH (2% BSII), in GOSH surveillance was predictive (30% versus 5%). Sensitivity was 33%–66% across sites and antibiotics. The rates of ESBL were higher in BSII from patients with preceding ESBL-producers on surveillance (31% versus 4% GOSH; 36% versus 3% AHCH). Extending the inclusion window to one year had minimal impact on sensitivity (<2% in both sites). Organism matching the prevalent species increased specificity at the expense of sensitivity, with increases in PPV. However, this was only a minimal improvement in predictive power.

**Conclusions:**

In two paediatric hospitals, the presence of resistance on surveillance was predictive of GN-BSII resistance across a range of antimicrobials. Predictive values indicate clinically significant differences. Predicting GN-BSII resistance by organism matching offered limited benefit, suggesting pattern of resistance is more informative than pathogen/resistance combinations. Gains by looking back beyond 6 months were limited. The similarity of results between two hospitals with different populations and surveillance practices is notable and may imply some generalizability. The application of these findings for empirical treatment in suspected sepsis will depend on clinical scenarios. Most episodes of suspected sepsis are blood culture negative, but many are caused by microorganisms resident in patient flora. Carriage of resistance is still expected to be relevant. Resistance in GI flora is relevant for predicting resistance in GN-BSII.

